# Effects of parental genetic divergence on gene expression patterns in interspecific hybrids of *Camellia*

**DOI:** 10.1186/s12864-019-6222-z

**Published:** 2019-11-08

**Authors:** Min Zhang, Yi-Wei Tang, Ji Qi, Xin-Kai Liu, Dan-Feng Yan, Nai-Sheng Zhong, Nai-Qi Tao, Ji-Yin Gao, Yu-Guo Wang, Zhi-Ping Song, Ji Yang, Wen-Ju Zhang

**Affiliations:** 1 Co-Innovation Center for Sustainable Forestry in Southern China, College of Biology and the Environment, Nanjing Forestry University, Nanjing, 210037 China; 2 Ministry of Education Key Laboratory for Biodiversity Science and Ecological Engineering, School of Life Sciences, Fudan University, Shanghai, 200438 China; 3 Palm Eco-Town Development Co, Ltd, Guangzhou, 510627 Guangdong China; 4 Research Institute of Subtropical Forest, Chinese Academy of Forestry, Fuyang, 311400 Zhejiang China

**Keywords:** *Camellia*, Allelic expression, Hybridization, Transcriptome shock, *Cis-* and *trans-* regulation

## Abstract

**Background:**

The merging of two divergent genomes during hybridization can result in the remodeling of parental gene expression in hybrids. A molecular basis underling expression change in hybrid is regulatory divergence, which may change with the parental genetic divergence. However, there still no unanimous conclusion for this hypothesis.

**Results:**

Three species of *Camellia* with a range of genetic divergence and their F_1_ hybrids were used to study the effect of parental genetic divergence on gene expression and regulatory patterns in hybrids by RNA-sequencing and allelic expression analysis. We found that though the proportion of differentially expressed genes (DEGs) between the hybrids and their parents did not increase, a greater proportion of DEGs would be non-additively (especially transgressively) expressed in the hybrids as genomes between the parents become more divergent. In addition, the proportion of genes with significant evidence of *cis*-regulatory divergence increased, whereas with *trans*-regulatory divergence decreased with parental genetic divergence.

**Conclusions:**

The discordance within hybrid would intensify as the parents become more divergent, manifesting as more DEGs would be non-additively expressed. *Trans*-regulatory divergence contributed more to the additively inherited genes than *cis*, however, its contribution to expression difference would be weakened as *cis* mutations accumulated over time; and this might be an important reason for that the more divergent the parents are, the greater proportion of DEGs would be non-additively expressed in hybrid.

## Introduction

Hybridization is an important power facilitating adaptive evolution [1]. In nature, hybridization is ubiquitous. It has been reported that over 25% of plant species and 10% of animal species are involved in hybridization or potential introgression with other species [2, 3]. Although most hybrids are infertile, some can possess novel phenotypic traits, like stronger stress tolerance and improved growth rate, which are better for their adaptation to hostile environments or expansion into new habitats; under natural selection, they also have the opportunity to evolve into new species [4–6].

Novel phenotypes can arise from changes of protein sequences. However, the variation of protein sequence is insufficient to explain so abundant morphological types present in nature [7]. Alternatively, the change of gene expression provides another source of phenotypic novelty [8]. There is growing evidence that merging of two divergent genomes during hybridization can result in the remodeling of parental gene expression patterns in hybrids, a phenomenon called “transcriptome shock” [9–12]. As manifestations, many genes would be non-additively expressed in hybrids (diverge from the mid-parental value), which contribute to their transgressive phenotypes at some extent [13, 14].

Broadly speaking, gene expression is controlled by the interactions between *cis*- and *trans*-acting elements, so transcriptome shock is likely in large part due to the variation of *cis*- and *trans*-regulation [15, 16]. *Cis*- and *trans*-regulatory divergence can be distinguished by measuring the allelic expression between two genotypes and their F_1_ hybrid. In F_1_ hybrid, two parental alleles are exposed to a common cellular environment, so *trans*-regulatory change has same effect on the two alleles, and their imbalanced expression is a readout of the relative *cis*-regulatory divergence [17]. Based on this strategy, a substantial effort has been made and revealed variable roles that *cis*- and *trans*-regulatory changes would play in reshaping gene expression. Previous studies on *Drosophila* showed that *cis*-regulatory change tended to result in the additive inheritance of gene expression [18, 19], but opposite result appeared in plant for that *trans*-regulatory change contributed more to the additive expression of the *Cirsium* hybrids [20]. In addition, the relative frequency of *cis*- and *trans*-regulatory divergence among studies was always inconsistent. Shi et al.’s study on *Arabidopsis* found that a greater proportion of genes showed significant evidence of *cis*- than *trans*-regulatory divergence [21], whereas Combes et al.’s study on *Coffea* got the opposite result [22]. Tirosh et al. found that *cis*-regulatory divergence seemed to be more common between than within species [16]. That means the divergence of regulatory patterns revealed by different works may be related to the genetic divergence of the parental species they used, and parental genetic divergence may have great effect on the regulation of gene expression patterns in hybrids [18, 23, 24]. To validate these hypotheses, three species of *Camellia* L, including *C. azalea* Z. F. Wei, *C. chekiangoleosa* Hu and *C. amplexicaulis* (Pit.) Cohen-Stuart as well as their F_1_ hybrids [*C. azalea* (*♀*) × *C. chekiangoleosa* (♂) and *C. azalea* (♀) × *C. amplexicaulis* (♂)] were used in this study to detect the influence of parental genetic divergence on gene expression and regulatory patterns in hybrids. Two crosses represent the intra- and inter-sectional hybridization of *Camellia*, respectively. Through RNA sequencing and allelic expression analysis, we are arming to investigate how *cis*- and *trans*-regulations change with parental genetic divergence as well as their effect on gene expression in hybrid.

## Results

### Sequencing and mapping

**Fig. 1:**
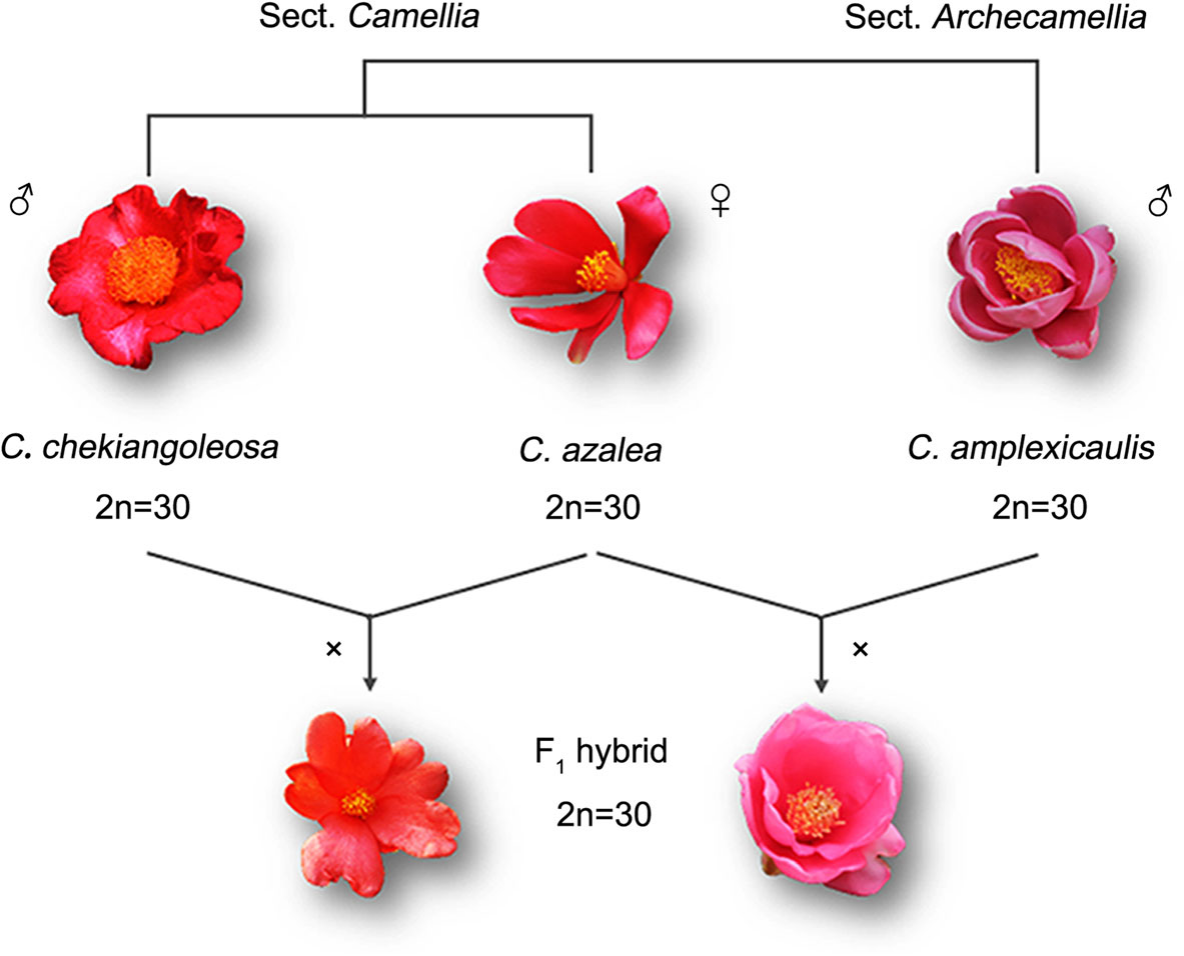
Diagram showing construction of the *Camellia* hybrids as well as materials used in this study

As described above, two crosses representing intra- and inter-sectional hybridization of *Camellia* were used in this study (Fig. 1). cDNA libraries were constructed using RNA extracted from flower buds of the F_1_ hybrids and their parental species, and then sequenced using the Illumina HiSeq X-ten platform. For each species and hybrid, three biologic replicates were set up. Finally, 664.6 million clean reads were obtained from 15 libraries with a mean of 44.3 million for each library. The proportion of clean reads with quality better than Q20 was over 97%, and better than Q30 was over 92% for each library (Additional file 1: Table S1). Three pseudo-genomes, representing the female and the two male parents, were constructed. Clean reads from the parental species were then realigned to their pseudo-genomes. The mean mapping rates for *C. azalea*, *C. chekiangoleosa* and *C. amplexicaulis* were ~ 70%. Clean reads from the hybrids were mapped to the pseudo-genomes of their parents, respectively. Although the mapping rates for the hybrids were relatively lower (~ 60%), we chose the maximum value of the two mapping results for each allele and their sum as the total reads count, which could counteract the influence of low mapping rates on the subsequent analysis.

### Changes of parental gene expression patterns in different F_1_ hybrids

**Fig. 2:**
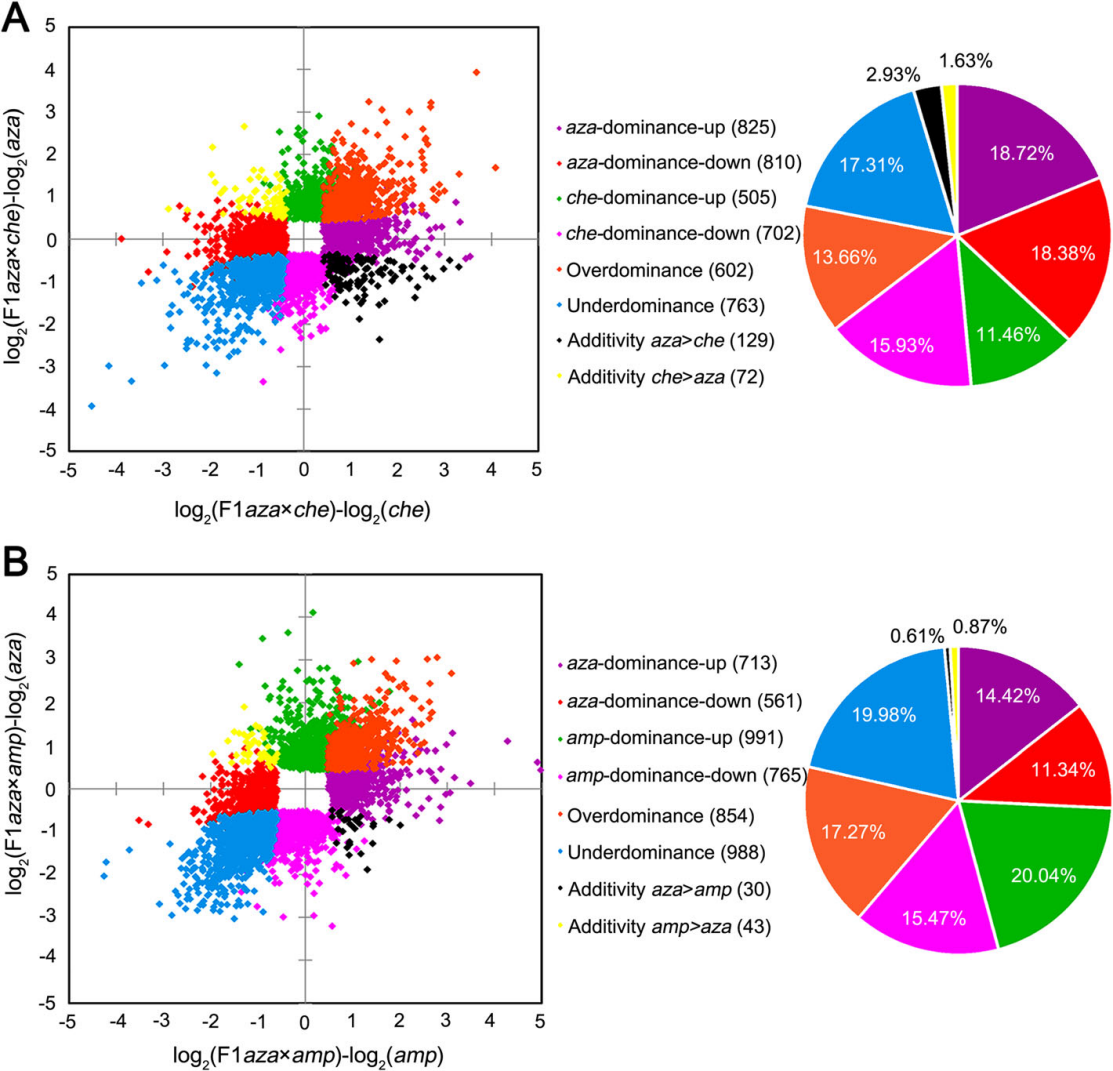
Classification of differentially expressed genes (DEGs) between the F_1_ hybrids and their parents. According to expression patterns, DEGs detected from the intra- (**a**) and inter-sectional (**b**) crosses were further classed into eight clusters as listed in the center of the images, respectively. Numbers in the brackets show genes included in each cluster, and pie charts show the relative proportions of DEGs for each cluster. *aza*, *Camellia azalea*; *che*, *C. chekiangoleosa*; *amp*, *C. amplexicaulis;* F1*aza × che*, F_1_ hybrid of *C. azalea* × *C. chekiangoleosa*; F1*aza × amp*, F_1_ hybrid of *C. azalea* × *C. amplexicaulis.* A fold-change of 1.25 combining with FDR < 0.05 were used as threshold for DEGs detection

Over half of the analyzed genes (57.8% for *C. azalea* × *C. chekiangoleosa* and 51.7% for *C. azalea* × *C. amplexicaulis*) were significantly differentially expressed between the F_1_ hybrids and at least one of their parents. Regardless of parental divergence, DEGs between the hybrids and their parents for each cross were further classified into eight clusters (Fig. 2). For the cross of *C. azalea* × *C. chekiangoleosa*, the relative proportion of genes belonging to additivity (including additivity female > male and female < male), female dominance (including dominance up and down), male dominance (including dominance up and down) and transgressivity (over-dominance and under-dominance) was 4.56, 37.09, 27.38 and 30.97%, respectively; while for the cross of *C. azalea* × *C. amplexicaulis*, the proportion was 1.48, 25.76, 35.51 and 37.25%, respectively. Compared with the intra-sectional cross (95.44%), a greater proportion of DEGs between the hybrids and their parents exhibited a non-additively expressed pattern in the inter-sectional cross (98.52%) (Fisher’s exact test, *P*-value < 2.2e^− 16^). The relative proportion of DEGs with transgressive expression pattern was significantly higher in the inter-sectional hybrid (37.25%) than that in the intra-sectional hybrid (30.97%) (Fisher’s exact test, *P*-value = 9.0e^− 11^). Pearson correlation analysis showed that the total expression level of the F_1_ hybrid of *C. azalea* × *C. chekiangoleosa* was more similar to its parents (*cor* > 0.81, *P*-value < 2.2e^− 16^) than the hybrid of *C. azalea* × *C. amplexicaulis* (*cor* < 0.79, *P*-value < 2.2e^− 16^) (Additional file 1: Figure S1).

### Allelic expression tests reveal *cis*- and *trans*-regulatory divergence in different crosses

**Fig. 3:**
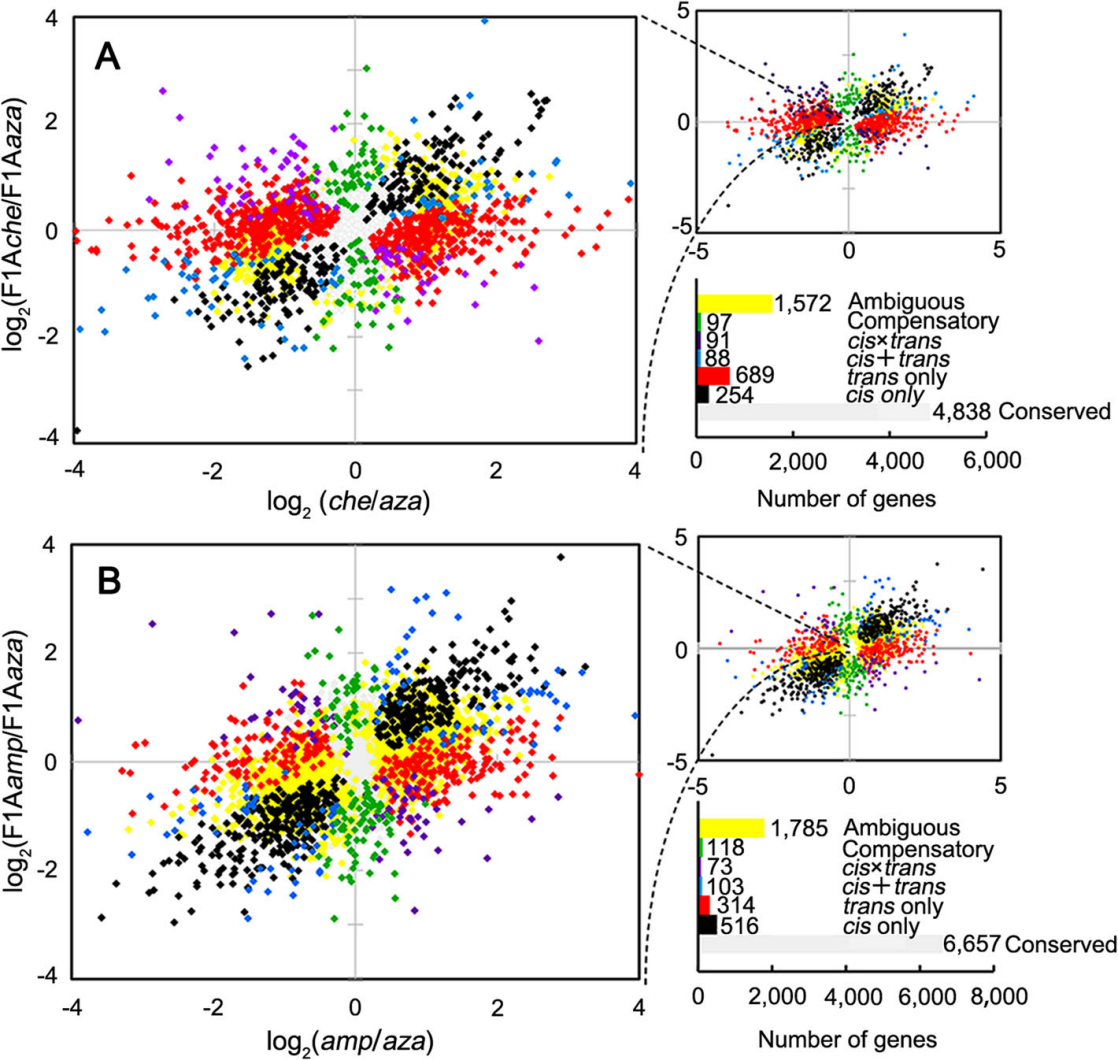
Plots summarize the relative allele-specific gene expression as well as gene regulation patterns in different crosses. **a** The cross of *Camellia azalea* × *C. chekiangoleosa*. **b** The cross of *C. azalea* × *C. amplexicaulis*. Each point represents a single gene and is color-coded according to the regulatory type (as shown in the bar graphs) it is regulated by. *aza*, *C. azalea*; *che*, *C. chekiangoleosa*; *amp*, *C. amplexicaulis*; F1A*aza*, allele from *C. azalea* in the F_1_ hybrid; F1A*che*, allele from *C. chekiangoleosa* in the F_1_ hybrid. F1A*amp*, allele from *C. amplexicaulis* in the F_1_ hybrid

Of the 7629 genes detected in the cross of *C. azalea* × *C. chekiangoleosa*, 8.09% (617) showed significant evidence of *cis*-regulatory divergence. When it came to the cross of *C. azalea* × *C. amplexicaulis*, the proportion of genes with significant evidence of *cis*-regulatory divergence was 10.31% (986 of 9566). Expression differences between species not attributable to *cis*-regulatory divergence could be caused by *trans*-regulatory divergence. In *C. azalea* × *C. chekiangoleosa*, 13.34% (1018 of 7629) of the genes showed significant evidence of *trans*-regulatory divergence, compared with 8.24% (629 of 9566) in *C. azalea* × *C. amplexicaulis*. There are 3.32% (254 of 7629) and 9.03% (689 of 7629) of genes in *C. azalea* × *C. chekiangoleosa* subjected to “*cis only*” and “*trans only*”, respectively. For *C. azalea* × *C. amplexicaulis*, these numbers become 5.39% (516 of 9566) and 3.28% (314 of 9566), respectively (Fig. 3). In addition, there were also 276 (3.62% of 7629) genes in *C. azalea* × *C. chekiangoleosa* and 294 (3.07% of 9566) genes in *C. azalea* × *C. amplexicaulis* showed significant evidence of both *cis*- and *trans*-regulatory divergence. Genes with significant evidence of both *cis*- and *trans*-regulatory divergence were further divided into three clusters, i.e., “*cis* + *trans*”, “*cis* × *trans*” and “compensatory” (Additional file 1: Table S2). The proportion of genes belong to the above three clusters in the cross of *C. azalea* × *C. chekiangoleosa* was 1.15% (88), 1.19% (91) and 1.27% (97), respectively; while in *C. azalea* × *C. amplexicaulis* was 1.08% (103), 0.76% (73) and 1.23% (118), respectively.

### Regulatory difference underling expression divergence between species

**Fig. 4:**
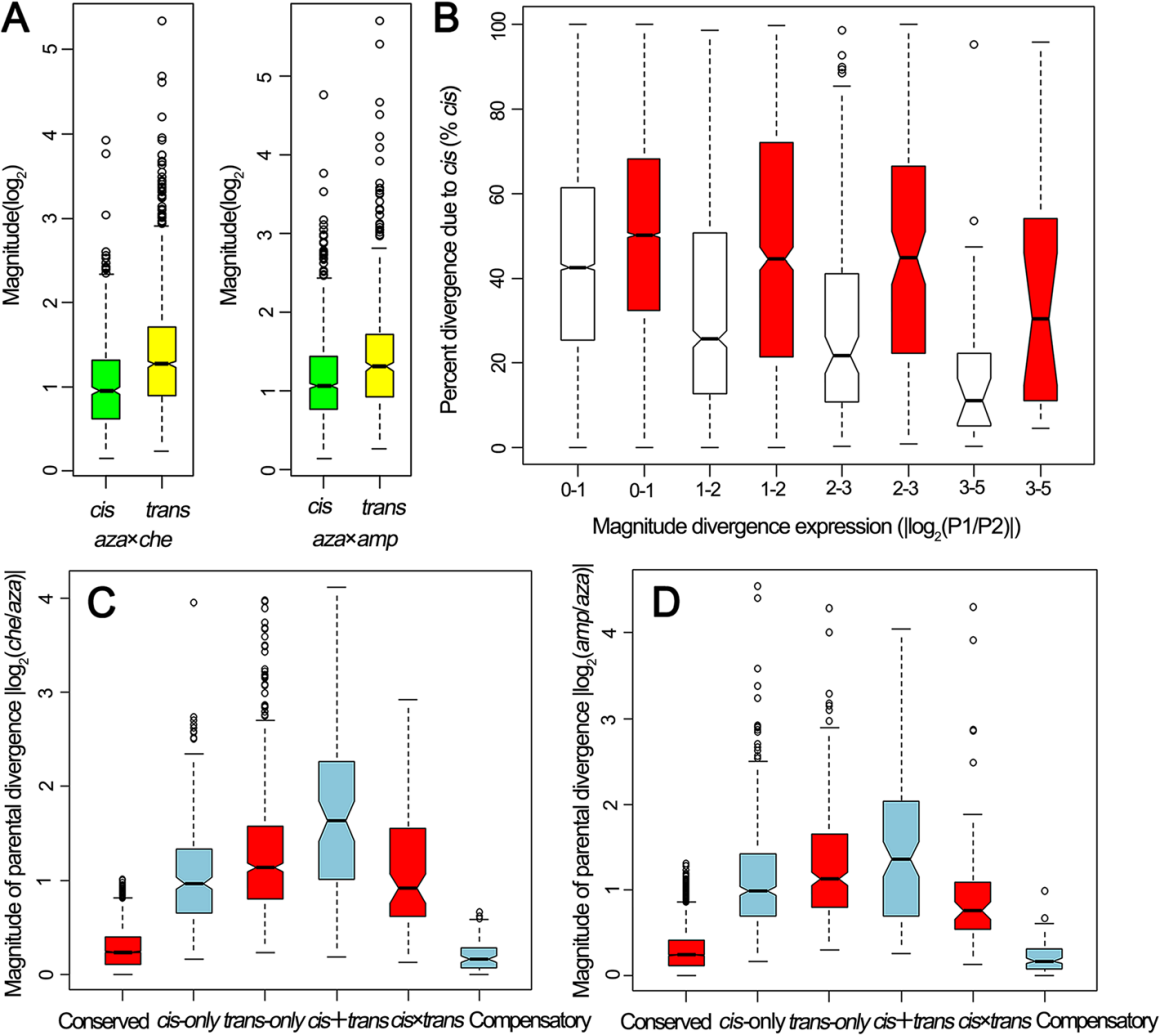
Influence of regulatory types on the expression divergence between the parental species. **a** Absolute magnitude (fold-change) of parental expression divergence resulting from *cis*- and *trans*-regulatory changes. *aza×che*, Comparison between *Camellia azalea* and *C. chekiangoleosa*; *aza×amp*, Comparison between *C. azalea* and *C. amplexicaulis*. **b** Percentage of total regulatory divergence attributable to *cis*-regulatory changes (% *cis*) for genes with different magnitudes of expression divergence between parents. P1, parent1; P2, parent2; Blank, comparison between *C. azalea* and *C. chekiangoleosa*; Red, comparison between *C. azalea* and *C. amplexicaulis*. **c** and **d** Absolute magnitude (fold-change) of parental expression divergence resulting from different regulatory types. *aza*, *C. azalea*; *che*, *C. chekiangoleosa*; *amp*, *C. amplexicaulis*

The median significant *trans*-regulatory difference between *C. azalea* and *C. chekiangoleosa* was 1.26 folds, which was significantly larger than the median *cis*-regulatory difference (0.94-fold, Wilcoxon’s rank-sum test, *P*-value < 2.2e^− 16^). Same pattern was also detected between *C. azalea* and *C. amplexicaulis* (Wilcoxon’s rank-sum test, *P*-value = 1.0e^− 15^), of which the median significant *trans*-regulatory difference was 1.30-fold, and the median significant *cis*-regulatory difference was 1.06-fold, respectively (Fig. 4a). Kendall’s test showed that, the expression differences between *C. azalea* and *C. chekiangoleosa* correlated more strongly with *trans*-regulatory divergence (*τ* = 0.34, *P*-value < 2.2e^− 16^) than with *cis*-regulatory divergence (*τ* = 0.12, *P*-value < 2.2e^− 16^). Same pattern was also detected between *C. azalea* and *C. amplexicaulis*, of which *trans*-regulatory divergence contributed more to the expression divergence (*τ* = 0.21, *P*-value < 2.2e^− 16^) than *cis*-regulatory divergence (*τ* = 0.18, *P*-value < 2.2e^− 16^). The amount of total regulatory divergence explained by *cis*-regulatory difference (% *cis*) decreased with the absolute magnitude of expression divergence between *C. azalea* and the other two species (Fig. 4b). However, the contribution of *cis*-regulatory difference to the expression divergence between *C. azalea* and *C. amplexicaulis* increased significantly compared with that between *C. azalea* and *C. chekiangoleosa* (Wilcoxon’s rank-sum test, *P*-value < 2.2e^− 16^). We also compared the absolute magnitude changes of parental expression divergence with different regulatory categories. As shown in Fig. 4c and d, “*trans* only” play a larger role than “*cis* only” in aggravating expression divergence between different species (Wilcoxon’s rank-sum test, *P*-value < 0.001). Furthermore, the interaction effect of *cis*- and *trans*-regulations functioning in the same direction (*cis* + *trans*) could tremendously change the gene expression patterns between two species. However, when the two regulations worked in the opposite direction (“*cis* × *trans*” and “compensatory”), the divergence of gene expression would be relieved to a large extent.

### Regulatory divergence underling gene expression patterns in different F_1_ hybrids

**Fig. 5:**
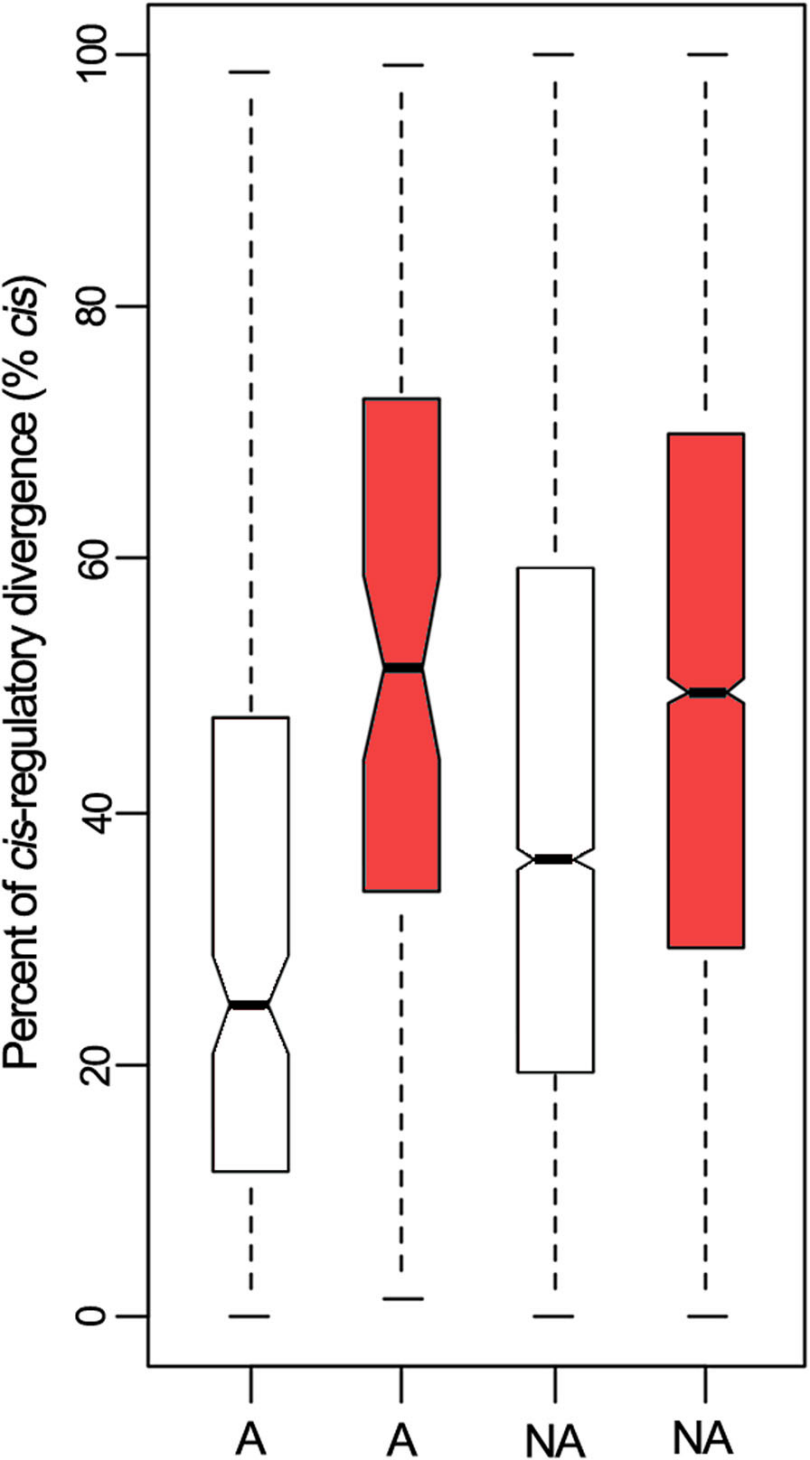
Percent of *cis*-regulatory divergence for genes showing additive and non-additive expression in *Camellia* F_1_ hybrids. A, additively expressed genes; NA, nonadditively expressed genes. Blank, F_1_ hybrid of *Camellia azalea* × *C. chekiangoleos*; Red, F_1_ hybrid of *C. azalea* × *C. amplexicaulis*

To examine the potential relationship between regulatory divergence and gene expression patterns in hybrid, we compared the % *cis* between sets of genes with additive and non-additive expression patterns in different hybrids. As shown in Fig. 5, in the F_1_ hybrid of *C. azalea* × *C. chekiangoleosa*, the median % *cis* for genes with non-additive expression patterns was significantly higher than that with additive expression patterns (Wilcoxon’s rank-sum test, *P*-value = 3.2e^− 7^). However, different result was detected in the hybrid of *C. azalea* × *C. amplexicaulis* for that there was no significant difference in the median % *cis* for additively and non-additively expressed genes (Wilcoxon’s rank-sum test, *P*-value = 0.1). In addition, % *cis* in the hybrid of *C. azalea* × *C. amplexicaulis* was significant higher than that in the hybrid of *C. azalea* × *C. chekiangoleosa* for either additively (Wilcoxon’s rank-sum test, *P*-value = 2.8e^− 8^) or non-additively inherited genes (Wilcoxon’s rank-sum test, *P*-value < 2.2e^− 16^). Most DEGs between the hybrids and their parents were subjected to the effects of “conserved” and “ambiguous”. Of the remaining DEGs with any expression patterns, a greater proportion were subjected to “*trans* only” than any other effects in the F_1_ hybrid of *C. azalea* × *C. chekiangoleosa*, while in the hybrid of *C. azalea* × *C. amplexicaulis*, a greater proportion were regulated by “*cis* only” (Table 1).

**Table 1:** Contributions of regulatory divergence to gene expression patterns in F_1_ hybrids

	*Camellia azalea × C. chekiangoleosa*				*C. azalea × C. amplexicaulis*			
	Additivity	Female dominance	Male dominance	Transgressivity	Additivity	Female dominance	Male dominance	Transgressivity
Conserved	0.00%	39.69%	40.93%	69.01%	0.00%	43.72%	52.56%	76.55%
Ambiguous	22.39%	33.27%	35.29%	18.40%	32.87%	34.23%	29.45%	16.01%
*Cis* only	13.43%	4.71%	6.79%	2.12%	36.99%	12.32%	8.71%	2.82%
*Trans* only	47.26%	18.17%	13.50%	5.71%	17.81%	6.51%	5.81%	2.01%
*Cis* + *trans*	14.43%	1.53%	1.33%	0.66%	12.33%	2.12%	1.82%	0.33%
*Cis* × *trans*	2.49%	1.96%	1.33%	1.54%	0.00%	0.86%	0.85%	0.71%
Compensatory	0.00%	0.67%	0.83%	2.56%	0.00%	0.24%	0.80%	1.57%

## Discussion

### Transcriptome shock in hybrid intensifies with parental genetic divergence

As described above, the merging of two divergent genomes during hybridization can result in “transcriptome shock”. Many studies reported the altered expression patterns in hybrids. Bell et al.’s study on the intraspecific hybridization of *Cirsium* found that 70.0% of the studied genes were differentially expressed between the F_1_ hybrid and at least one of its parents, of which 92.5% were non-additively expressed [20]. Combes et al.’s study on the interspecific hybridization of *Coffea canephora × C. eugenioides* found that DEGs between hybrids and the parents accounted for ~ 27% of the studied genes, of which 87.1% presented a non-additive pattern [22]. While for the study of *Drosophila melanogaster* and *D. sechellia*, the percent was 96%, of which 84% were non-additively expressed [19]. When it come to our study, ~ 50% of the genes were differentially expressed between the hybrids and at least one of their parents in either the intra-sectional or the inter-sectional hybridization, and most of them were non-additively expressed in the hybrids (Fig. 2). Based on the fragments which are available at NCBI and widely used for phylogenetic analysis (Additional file 1: Table S3), we calculated the genetic distances between the parental species of different studies. Regardless of the intraspecific hybridization of *Cirsium*, genetic distance between *C. canephora* and *C. eugenioides* is 0.025, between *D. melanogaster* and *D. sechellia* is 0.048, while between *C. chekiangoleosa, C. amplexicaulis* and *C. azalea* are 0.025 and 0.050, respectively. We found there are no linear relationship between the percent of DEGs and the parental genetic distance. A potential reason for this maybe that these works were conducted under different experimental systems. However, in our study, under the same experimental system, we found that the percent of DEGs between the hybrids and their parents did not increase linearly as genetic distance between the parents become bigger, too. This seems doesn’t meet our expectation that the more divergent the parents are, the greater proportion of genes would be differentially expressed between the offspring and the parents. In fact, Coolon et al. also found that the DEGs did not increase consistently with divergence time, and they speculated that increasing magnitudes of expression differences rather than increasing numbers of genes with divergent expression drive the overall increase in expression differences with divergence time [24]. A potential model may be that, in a definite scope, DEGs between hybrids and their parents would increase with parental genetic distance. However, beyond this scope, new pattern may appear. Our results support this hypothesis. In our study, although the proportion of DEGs decreased to some extant in the inter-sectional hybrid, a greater proportion of DEGs would be non-additively expressed in the inter-sectional hybrid than that in the intra-sectional hybrid. Specifically, more DEGs were transgressively expressed in the inter-sectional hybrid than that in the intra-sectional hybrid. That means the relative proportion of non-additively (especially transgressively) expressed gene within DEGs in hybrids would increase with parental genetic divergence. Correspondingly, the total expression level of genes in the inter-sectional hybrid was more diverge from its parents than that in the intra-sectional hybrid as shown in Additional file 1: Figure S1. These results could serve as important evidence that transcriptome shock in hybrid would intensify with parental genetic divergence.

### Relative frequency of *cis*- and *trans*-regulatory divergence in different hybrids

According to previous studies, *cis*- and *trans*-regulatory divergence have their own ways in affecting gene expression [19]. So, the relative frequency of *cis*- and *trans*-regulatory divergence has great influence on the inheritance of gene expression patterns in hybrid [18]. The relative frequency of *cis*- and *trans*-regulatory divergence revealed by different studies is always variable. Taking *Drosophila* for example, McManus et al.’s study on the hybrids of *D. melanogaster* × *D. sechellia* found that more genes showed significant evidence of *trans*- than *cis*-regulatory divergence [19]. In plants, Combes et al.’s study on *Coffea canephora* × *C. eugenioides* and Bell et al.’s study on the intraspecific hybridization of *Cirsium*, also found more genes were subjected to *trans*-regulatory divergence [20, 22]. However, when it came to the interspecific hybridization of *Arabidopsis thaliana* × *A. arenosa* more genes were significantly influenced by *cis*- rather than *trans*- regulatory divergence [21]. Denver et al. speculated that natural selection would eliminate most *trans*-acting mutations and accumulate *cis*-regulatory mutations over time [25]. That means the relative frequency of *cis*- and *trans*-regulatory changes in hybrids may be related to the divergence time between the parental species. To validate this inference, we calculated the genetic distances of the parental species involved in different studies. According to the nrDNA fragments, the genetic distance between *D. melanogaster* and *D. sechellia* is 0.048, between *C. canephora* and *C. eugenioides* is 0.025, while between *Arabidopsis thaliana* and *A. arenosa* is 0.050. According to these data, *cis*-regulatory changes tend to be dominant when the parental genetic distance is enough big.

When it came to our study, the *cis*- and *trans*-regulatory divergences in different crosses were distinguished using the same method with unified criterions. However, the results were completely different for that the proportions of genes with significant evidence of *cis*- and *trans*-regulatory divergence in the intra-sectional cross (*C. azalea* × *C. chekiangoleosa*) were 8.09 and 13.34%, respectively, whereas in the inter-sectional cross of *C. azalea* × *C. amplexicaulis* were 10.31 and 8.24%, respectively. In other words, *trans*-regulatory divergence was more prevailing than *cis*- in the intra-sectional cross, while in the inter-sectional cross was just the opposite. These results indicate that the proportion of genes with significant evidence of *cis*-regulatory divergence would increase, while with significant evidence of *trans*-regulatory divergence would decrease with genetic divergence between species. A potential reason for this phenomenon may be that *cis*-regulatory mutations are more likely to be fixed than *trans*- under natural selection. This seems to be inconsistent with a neutral model assuming equal probabilities of fixation for *cis*- and *trans*-regulatory polymorphisms. In fact, *cis*-acting mutations in the promoter region may simply alter the transcript levels of gene(s) downstream, whereas a *trans*-acting mutation in a transcription factor may result in multiple downstream regulatory changes [26]. For selection, it must act more strongly against mutations with pleiotropic effects to operate more efficiently [27]. So *trans*-regulatory mutations with multiple effects are more likely to be eliminated. Specifically, as Wittkopp et al. [28] speculated, *trans*-acting mutations may include both highly pleiotropic changes as well as some with limited effects, the former ones are more likely to be eliminated, while the later ones could be accumulated by mutation-selection balance. This may be an important pattern for the evolution of *cis*- and *trans*-regulation.

### *Cis*- and *trans*-regulatory differences underlying expression divergence between species

McManus et al.’s study on the hybrid of *Drosophila* showed that the median significant of *trans*-regulatory divergence was larger than that of *cis*-regulatory divergence between species, and *trans*-regulatory divergence correlated more highly with the expression difference between species [19]. Same profile also appeared in the study of *Cirsium* [20]. Similarly, our results showed that *trans*-regulatory change contributed more to the expression divergence between *C. azalea* and the other two species than *cis*-regulatory change. Correspondingly, the expression differences between *C. azalea* and the other two species correlated more highly with *trans*-regulatory changes, too. That means *trans*-regulatory change plays a larger role than *cis*-regulatory change in promoting the differentiation of gene expression between species. We also detected the changes of the % *cis* with the absolute magnitude of total expression divergence between species. As shown in Fig. 4b, the relative percent of *cis*-regulatory divergence decreased with the absolute magnitude of total expression divergence between *C. azalea* and the other two species. In other words, genes which were more deeply affected by *trans*-regulatory change would be more divergently expressed between species. This was consistent with the result generated from previous studies [20, 24]. However, as *cis*-regulatory mutation accumulates over time, its influence on expression divergence increases, too. This could be deduced from the fact that the contribution of *cis*-regulatory change (% *cis*) to the expression divergence between *C. azalea* and *C. amplexicaulis* at any level was obviously higher than that between *C. azalea* and *C. chekiangoleosa* (Fig. 4b).

*Cis*- and *trans-*regulatory divergence are not mutually exclusive, many genes would be significantly influenced by both *cis*- and *trans*-regulatory changes [19, 20, 22, 24, 29]. In our study, 3.62% of the studied genes between *C. azalea* and *C. chekiangoleosa* and 3.07% between *C. azalea* and *C. amplexicaulis* showed significant evidence of both *cis*- and *trans*-regulatory divergence. Interactions between *cis*- and *trans*-regulatory divergences can result in quite divergent expression patterns between species. As shown in Fig. 4c and d, *cis*- and *trans*- regulatory changes promoting expression of the same allele (*cis* + *trans*) could greatly stimulate the expression divergence between species. Conversely, if two regulatory categories act on the alternate alleles (*cis* × *trans*), the divergence of gene expression between species would be relieved to some extent. Specifically, the compensatory effect of *cis*- and *trans*-regulatory changes tended to eliminate expression divergence between species. These findings are consistent with the results generated from *Coffea* [22] and *Arabidopsis* [21]. According to previous studies, genes significantly influenced by “*cis* + *trans*” might be driven by directional selection [16], whereas regulated by “*cis* × *trans*” as well as “compensatory” are likely to be driven by stabilizing selection [30]. In our study, the proportion of genes with significant evidence of both *cis*- and *trans*-regulatory divergence was lower in the cross of *C. azalea* × *C. amplexicaulis* than that in *C. azalea* × *C. chekiangoleosa*, mainly because of that fewer genes were affected by “*cis* × *trans*” (Fig. 3). This may just reflect the evolutionary history of the three *Camellia* species: *C. azalea* and *C. chekiangoleosa*, as two closely related species, have more genes experienced stabilizing selection; while for species from two divergent sections (*C. azalea* and *C. amplexicaulis*), fewer genes between them are driven by stabilizing selection.

### Contribution of regulatory divergence to gene expression patterns in hybrid

As described above, molecular basis underlying gene-expression difference is the variation of *cis*- and *trans*-regulations. Previous studies on *Drosophila* [19] and yeast [31] showed that *cis*-regulatory divergence appeared to result in additive inheritance of gene expression more often than *trans*-regulatory divergence. However, latest studies based on transcriptome analysis reported the opposite result, for that *trans*-regulatory divergence in these studies accounted for a greater proportion of the regulatory divergence at sites with additive than that with non-additive inheritance patterns [20, 24]. In our study, in the F_1_ hybrid of *C. azalea* × *C. chekiangoleosa*, the median of % *trans* was significant higher for genes showing additive expression pattern than that showing non-additive expression pattern (Fig. 5). However, in the hybrid of *C. azalea* × *C. amplexicaulis*, there was no significant difference in the relative percent of *cis*- and *trans*-regulatory divergence for neither additively nor non-additively expressed genes. We speculate that the relative contribution of *cis*- and *trans*-regulatory divergence (% *cis* and % *trans*) to the inheritance of gene expression may depend on the parental genetic divergence. A potential mode is that *trans*-regulatory divergence is more likely to lead to additive inheritance than *cis*-regulatory divergence. For hybrids whose parents are closely related species, the relative frequency of *trans*-regulatory divergence is higher than that of *cis*-regulatory divergence; however, as genomes between the two parents become more divergent, *trans*-regulatory mutations are eliminated to some extent and *cis*-regulatory divergence becomes dominant. This could be used to explain why a higher proportion of genes would be non-additively (especially transgressively) expressed in the F_1_ hybrid of inter-sectional than that of intra-sectional hybridization.

The interactions between *cis*- and *trans*-regulatory divergences can greatly affect gene expression patterns between species. There were studies showed that “*cis* × *trans*” regulatory divergence was more common in transgressively expressed genes [19, 29]. However, study on the hybrids of *Cirsium* found that genes with transgressive expression pattern were mainly regulated by “*cis* + *trans*” [20]. In our study, neither “*cis* × *trans*” nor “*cis* + *trans*” regulation was the major reason leading to the transgressive expression patterns in hybrids (Table 1). Instead, most of the DEGs between the hybrids and their parents followed a “conserved” or “ambiguous” manner. In addition, compared with the intra-sectional hybridization, a greater proportion of DEGs were subjected to “*cis* only” effect in the inter-sectional hybridization for any expression patterns. So, inheritance of gene expression patterns is more likely to be the result of the comprehensive effects of different regulatory mechanisms, and the change of relative frequency of *cis*- and *trans*-regulatory divergence plays an important role in the formation of divergent expression patterns in hybrid.

## Conclusions

In this study, by comparing the gene expression patterns between the *Camellia* hybrids and their parents, we found that the relative proportion of DEGs with non-additively expressed patterns in hybrid would increase with parental genetic divergence, which indicated that the discordance within hybrid would intensify as the parents become more divergent. Meanwhile, the proportion of genes with significant evidence of *cis*-regulatory divergence increased, while with *trans*-regulatory divergence decreased with parental genetic divergence. *Trans*-regulatory change contributes more to the additively inherited genes in hybrid than *cis*-. So, the weakening of *trans*-regulatory effect and the strengthen of *cis*-regulatory effect provide a major reason for the phenomenon that the more divergent the parents are, the greater proportion of DEGs would be non-additively expressed in hybrid.

## Methods

### Plant materials and hybridization

Three species including *C. azalea* (2n = 30), *C. chekiangoleosa* (2n = 30) and *C. amplexicaulis* (2n = 30) were used in this study (Fig. 1). According to morphological and molecular studies, both *C. azalea* and *C. chekiangoleosa* belong to the Sect. *Camellia* of *Camellia*, while *C. amplexicaulis* belongs to the Sect. *Archecamellia* of *Camellia* [32, 33]. Hybridizations were carried out by Palm Eco-Town Development Co. Ltd. in 2007 following the technique described by Gao et al. [34]. For all the hybridization experiments, *C. azalea* was served as the female parent, and the other two species were served as the male parents. All the plants in this study are grown in a same greenhouse of Palm Eco-Town Development Co. Ltd. at Guangzhou, China. To improve pollination efficiency, pollens from different individuals of the two paternal species were collected together, respectively. The mixed pollens were then used to pollinate the flowers of *C. azalea* plants. So, the F_1_ hybrids may be not from the identical parents, but their parents came from individuals of one wild population, respectively. Finally, two F_1_ hybrid series, *C. azalea* (♀) × *C. chekiangoleosa* (♂) and *C. azalea* (♀) × *C. amplexicaulis* (♂), representing intra-sectional and inter-sectional hybrids, were successfully obtained. Flower buds of the F_1_ hybrids and their parents at same stage were harvested and frozen in liquid nitrogen immediately, then transferred to − 80 °C refrigerator for storage.

### RNA extraction and sequencing

Total RNA was extracted from the flower buds using the RNAprep Puree Plant Kit DP441 (TIANGEN, Beijing, China) according to the manufacturer’s instructions. For each species and hybrid, three biologic replicates (from three individuals, respectively) were set up as parallel experiments. Nanodrop 1000 spectrophotometer (Thermo Fisher Scientific, Wilmington, DE, USA) was used to detect the quantity and quality of RNA. RNA-seq library was constructed for each sample. In total, 15 libraries were constructed, and then paired-end (2 × 150 bp) sequenced using Illumina HiSeq X-ten platform (Illumina Inc., San Diego, CA, USA) by Beijing Genomics Institute (BGI, Shenzhen, China) with the standard Illumina RNA-seq protocols.

### Mapping transcriptome reads to the reference genome

Clean reads were obtained by removing reads with adapter contamination and ploy-Ns (≥ 5%) as well as low quality reads with over 20% of low-quality bases (Phred < 15). Since the reference genomes of the species in our study were not available, filtered reads from the parental libraries were first mapped to the genome of *C. sinensis var. assamica* [35] using STAR software [36] with default parameters, and only uniquely mapped reads were retained. According to the ITS sequences (Additional file 1: Table S3), the genetic distances between *C. azalea*, *C. chekiangoleosa*, *C. amplexicaulis* and *C. sinensis* was 0.044, 0.045 and 0.046, respectively. Though with genetic divergence at some extant, according to Vijayan ‘s study [33], they are still closely related species. So, *C. sinensis* is appropriate as reference for the RNA-seq analysis. Then, SAMtools [37] and VarScan [38] software were orderly used for SNP calling. SNP sites at which three replicates were consistent were marked. For allelic expression research, a tough problem deserving consideration is mapping bias. To relieve mapping bias, three pseudo-genomes, representing the female and the two male parents, were constructed by replacing the reference alleles in the *C. sinensis* genome with the corresponding alternative alleles at the SNP sites, respectively. Then, transcriptome reads from the parental libraries were realigned to their pseudo-genomes using the same parameters to obtain the final read counts at the SNP sites. When it came to the F_1_ hybrid series, reads from each library were mapped to the two pseudo-genomes of their parents, respectively. To relieve mapping bias, for each parental allele in the hybrids, we chose the maximum value of the two mapping results as the final reads count at each SNP site, and the sum of the two alleles as the total reads count at one site. To identify reliable SNP sites, quality controls were applied as follows: (i) the SNP sites in the two parents must be homozygous for difference; (ii) each SNP site in the F_1_ hybrid must consist of only two alleles (one for the male parent, another for the female parent); (iii) the read count of the minor parental allele in the hybrid at each SNP site must be ≥2; and (iv) the total read count at each SNP site must be ≥20.

### Gene expression quantitation

We wrote a R script to identify species-specific SNP sites from the mapping results. Finally, 37,078 SNPs, representing 7629 genes were identified from the cross of *C. azalea* × *C. chekiangoleosa*; and 81,477 SNPs, representing 9566 genes, were identified from the cross of *C. azalea* × *C. amplexicaulis.* Transcript abundances of genes were evaluated as the normalized reads mapped per SNP site. Trimmed Mean of M-values (TMM) method [39] implemented in the edgeR package [40] was used for data normalization across libraries based on the assumption that most genes are not differentially expressed. Gene expression level was independently quantified for each cross, taking the biological replicates into consideration. The normalized gene expression for each cross is provided in the supporting information (Additional file 1: Tables S4 and S5). Cluster analysis was then carried out to examine the repeatability of the three biological replicates. As shown in Additional file 1: Figure S2, nearly all the biological replicates for each species and hybrid were clustered together (with *R*^2^ > 0.90) except for amp1 and aza_che3, and these two samples were removed in the following analyses.

### Classification of gene expression patterns

The edgeR package [40] was used for pairwise expression comparison, taking the three biological replicates into consideration. A fold change of 1.25 and the FDR < 0.05 were used as thresholds for differentially expressed gene (DEG) identification. DEGs between the hybrids and their parental species were further classed into eight clusters according to previous studies. Specifically, DEGs whose expression in the hybrid were higher than one of the parents but lower than another were classified as additivity (including additivity male > female and male < female); DEGs which were up/down-regulated in the hybrid compared with one of the parents but not differentially expressed with another were classified as male/female expression level dominance-up/down; DEGs whose expression level in the hybrid were significantly higher/lower than both of the parents were classified as transgressivity (overdominance/underdominance).

### Allelic expression patterns and *cis*- and *trans*-regulatory divergence assignment

Based on the species-specific SNP information, relative expression of the parental alleles in hybrids was evaluated. For each allele, the mean value of the three biological replicates was used for allelic expression as well as the subsequent regulatory divergence analysis. Expression divergence between the parental species is mainly caused by the combination of *cis*- and *trans*-regulatory changes, which could be quantified as log_2_ (parent1/parent2). In F_1_ hybrid, two parental alleles are exposed to a common *trans*-regulatory environment, and are equally affected by the *trans*-regulatory change. So, the log_2_-transformed radio of allelic expression in hybrid was used to quantify the degree of *cis*-effect: *cis* = log_2_ (F_1_A_parent1_/F_1_A_parent2_). Binomial exact test with FDR correction (FDR: 5%) was used to determine the significant *cis*-effect with a null hypothesis F_1_A_parent1_ = F_1_A_parent2_. Then *trans*-regulatory divergence was calculated as the difference between log_2_-transformed ratios of species-specific reads in the parents and the hybrids: *trans* = log_2_(parent1/parent2) - log_2_(F_1_A_parent1_/F_1_A_parent2_). Fisher’s exact test with FDR corrections (FDR: 5%) was used to identify the statistically significant *trans*-effects with a null hypothesis parent1/parent2 = F_1_A_parent1_/ F_1_A_parent2_. The relative proportion of total regulatory divergence attributable to *cis*-regulatory divergence (% *cis*) was calculated as (% *cis*) = [|*cis*|/(|*cis*| + |*trans*|)] × 100%, similarly, % *trans*. In addition, binomial exact test (FDR: 5%) was used to detect the significantly different expression between the two parental species. Regulatory divergence for different genes was then identified based on the results of binomial and Fisher’s tests as well as the direction of changes. According to previous studies [24], seven regulatory types were further identified (Additional file 1: Table S2). (i) *cis* only: the parental alleles were unequally expressed in the same ratio in F_1_ hybrid and between the two parents. (ii) *trans* only: the parental alleles were equally expressed in F_1_ hybrid but unequally expressed between the two parents. (iii) *cis* + *trans*: the parental alleles were unequally expressed both in F_1_ hybrid and between the two parents, but have the same direction (species with higher expression contributed the higher expressing allele in the F_1_ hybrid). (iv) *cis* × *trans*: the parental alleles are unequally expressed both in F_1_ hybrid and between the two parents, but have the opposite direction (species with higher expression contribute the lower expressing allele in the F_1_ hybrid). (v) Compensatory: the two parental alleles are equally expressed between the two parents but unequally in the F_1_ hybrid. (vi) conserved: the parental alleles are equally expressed both between the two parents and within the F_1_ hybrid. (vii) ambiguous: other situations not included in the above six categories.

### Statistical test

Pearson correlation analysis was carried out to detect the relationship of gene expression between the hybrids and their parents. Wilcoxon’s rank-sum test was performed to compare the median parental expression divergence attributable to *cis* and *tran*s-regulation. Kendall’s test was used to detect the relative contribution of *cis*- and *trans*-regulation to the divergent gene expression between different species. All the test statistics were calculated in R programe (v 3.3.2, CRAN). The main scripts used in this study are available in the supporting information.

## Supplementary information

**Additional file 1: Figure S1.** Plots compare total expression levels of F_1_ hybrids to total expression levels of parental species. *aza*, *Camellia azalea*; *che*, *C. chekiangoleosa*; *amp*, *C. amplexicaulis*; F1*aza × che*, F_1_ hybrid of *C. azalea* × *C. chekiangoleosa*; F1*aza × amp*, F_1_ hybrid of *C. azalea* × *C. amplexicaulis*. **Figure S2.** Clustering analysis showing the repeatability of biological replicates for each species and the hybrid. **(A)** The cross of *Camellia azalea* × *C. chekiangoleosa*. **(B)** The cross of *Camellia azalea* × *C. amplexicaulis*. Samples started with *aza*, *C. azalea*; *che*, *C. chekiangoleosa*; *amp*, *C. amplexicaulis*. *aza_che*, F_1_ hybrid of *Camellia azalea* × *C. chekiangoleosa*; *aza*_*amp*, F_1_ hybrid of *Camellia azalea* × *C. amplexicaulis*. **Table S1.** The sequencing results of different accessions in this study**. Table S2.** Classification of different regulatory types. **Table S3.** Sequences used for genetic distance analysis in this study. **Table S4.** The normalized gene expression of the intra-sectional cross. **Table S5.** The normalized gene expression of the inter-sectional cross.

## Data Availability

All the sequencing data is available at the NCBI Sequence Read Archive (SRA) database with the accession number of SRP144621.

## Abbreviation

DEGs: Differentially expressed genes
