# Supplementary material for: Effects of parental genetic divergence on gene expression patterns in interspecific hybrids of Camellia
Source: BMC Genomics. 2019 Nov 8;20:828. doi: 10.1186/s12864-019-6222-z (PMC6842218; doi:10.1186/s12864-019-6222-z)
Supplement: Supplementary file 1 — Additional file 1: Figure S1. Plots compare total expression levels of F1 hybrids to total expression levels of parental species. aza, Camellia azalea; che, C. chekiangoleosa; amp, C. amplexicaulis; F1aza × che, F1 hybrid of C. azalea × C. chekiangoleosa; F1aza × amp, F1 hybrid of C. azalea × C. amplexicaulis. Figure S2. Clustering analysis showing the repeatability of biological replicates for each species and the hybrid. (A) The cross of Camellia azalea × C. chekiangoleosa. (B) The cross of Camellia azalea × C. amplexicaulis. Samples started with aza, C. azalea; che, C. chekiangoleosa; amp, C. amplexicaulis. aza_che, F1 hybrid of Camellia azalea × C. chekiangoleosa; aza_amp, F1 hybrid of Camellia azalea × C. amplexicaulis. Table S1. The sequencing results of different accessions in this study. Table S2. Classification of different regulatory types. Table S3. Sequences used for genetic distance analysis in this study. Table S4. The normalized gene expression of the intra-sectional cross. Table S5. The normalized gene expression of the inter-sectional cross. [file 12864_2019_6222_MOESM1_ESM.zip › Additional file.docx]

**Additional file 1：**

**Effects of parental genetic divergence on gene expression patterns in interspecific hybrids of *Camellia***

Min Zhang^1,2^, Yi-Wei Tang^2^, Ji Qi^2^, Xin-Kai Liu^3^, Dan-Feng Yan^3^, Nai-Sheng Zhong^3^, Nai-Qi Tao^2^, Ji-Yin Gao^3,4^, Yu-Guo Wang^2^, Zhi-Ping Song^2^, Ji Yang^2^, Wen-Ju Zhang^2,*^

^1^ College of Biology and the Environment, Nanjing Forestry University, Nanjing 210037, China

^2^ Ministry of Education Key Laboratory for Biodiversity Science and Ecological Engineering, School of Life Sciences, Fudan University, Shanghai 200438, China.

^3^ Palm Eco-Town Development Co, Ltd, Guangzhou, Guangdong 510627, China.

^4^ Research Institute of Subtropical Forest, Chinese Academy of Forestry, Fuyang, Zhejiang 311400, China.

* Corresponding author

Name: Wen-Ju Zhang, Tel: +86 21-51630707, E-mail: [wjzhang@fudan.edu.cn](mailto:wjzhang@fudan.edu.cn)

**Main script used in this study.**

## 1.Mapping and SNP calling run for every fastq pairs (shell scripts)

source /mnt/.bashrc

## mapping reads to reference genome

mkdir outputTEA

STAR --runMode genomeGenerate --runThreadN 15 --genomeDir ./outputTEA --genomeFastaFiles Teatree_Assembly.fas --sjdbGTFfile Teatree.gtf --sjdbOverhang 149;

STAR --runThreadN 15 --genomeDir ./outputTEA --readFilesIn reads_1.fq reads_2.fq --outFileNamePrefix xxresult --outFilterScoreMin 255;

samtools view -bS resultAligned.out.sam > resultAligned.bam

samtools sort -o resultAligned.sort.bam -@ 20 resultAligned.bam

samtools index resultAligned.sort.bam

samtools mpileup -f ../Teatree_Assembly.fas resultAligned.sort.bam > mileup.txt

java -jar /mnt/software/small_tools/VarScan.v2.3.9.jar mpileup2snp mileup.txt --min-coverage 20 --min-reads2 2 --min-var-freq 0.95 > outputxx.txt

## 2. Pseudo-genomes construction. After that, the reads should be mapped to the Pseudo-genomes repeatedly to gain a more accurate result. (R scripts)

library(seqinr)

data=read.fasta("Teatree_Assembly.fas")

vcf1=read.table("outputxx-1.txt",header=TRUE,stringsAsFactors=FALSE,sep="\t",quote="")

vcf2=read.table("outputxx-2.txt",header=TRUE,stringsAsFactors=FALSE,sep="\t",quote="")

vcf3=read.table("outputxx-3.txt",header=TRUE,stringsAsFactors=FALSE,sep="\t",quote="")##three repetitions

a1=apply(vcf1[,1:4],1,function(x){paste(x,collapse="_")})

a2=apply(vcf2[,1:4],1,function(x){paste(x,collapse="_")})

a3=apply(vcf3[,1:4],1,function(x){paste(x,collapse="_")})

a=intersect(a1,intersect(a2,a3))

vcf=vcf1[which(a1%in%a),1:4]

reads1=matrix(unlist(strsplit(vcf1[which(a1%in%a),5],split=":")),nrow=length(a),byrow=TRUE)

reads2=matrix(unlist(strsplit(vcf2[which(a2%in%a),5],split=":")),nrow=length(a),byrow=TRUE)

reads3=matrix(unlist(strsplit(vcf3[which(a3%in%a),5],split=":")),nrow=length(a),byrow=TRUE)

clean=c("sc","position","ref","alter","depth_s1","clean_s1","depth_s2","clean_s2","depth_s3","clean_s3")

result=data

for(i in 1:length(a)){

test=min(as.numeric(reads1[i,4])/as.numeric(reads1[i,2]),as.numeric(reads2[i,4])/as.numeric(reads2[i,2]),as.numeric(reads3[i,4])/as.numeric(reads3[i,2]))

if(test>0.85){

clean=rbind(clean,c(vcf[i,],reads1[i,c(2,4)],reads2[i,c(2,4)],reads3[i,c(2,4)]))

result[[vcf[i,1]]][vcf[i,2]]=vcf[i,4]}

}

write.table(clean,"CLEAN-VCF-xx.txt",row.names=FALSE,col.names=FALSE,quote=FALSE,sep="\t")

write.fasta(result,names(result),"concensus-x")

## Constructing Pseudo-genome for each species

## For the hybrids, reads were mapped to their parents, respectively.

## 3. Read counts calling for each SNP site. (shell scripts)

## head -n 1 CLEAN-VCF-xx.txt >header.txt;

sed -i '1d' CLEAN*; ##delete header

cut -f 1,2,4 CLEAN* > xxsnp_loc.txt;##CHOOSE SNP LOCATION COLUMNS

sort xxsnp_loc.txt | uniq -u > xxsnp_loc.sort.txt;##choose parentally specific part

cut -f 1,2 xxsnp_loc.sort.txt | sort -u > xxfinal_snp_loc.txt;##remove redundant loci and get snp loci

sed 's/$/\t/g' xxfinal_snp_loc.txt > xxfinal_snp_loc.parallelinput;

java -jar /mnt/software/small_tools/VarScan.v2.3.9.jar mpileup2cns xx-mileup.txt --min-coverage 20 --min-reads2 2 --min-var-freq 0.95 >output-xx-cns.txt;

cut -f 1,2,3,4,5 output-xx-cns.txt > output-xx-cns.cut.txt;

cat output-xx-cns.cut.txt | parallel --block 20M --pipe grep -Ff xxfinal_snp_loc.parallelinput > xx-snp_expression.txt;

## 4. Filter the snps and calculate the gene expression (R scripts)

list=list.files()

list=grep("xx-snp_expression.txt",list,value=TRUE)

data0=read.table(list[1],header=FALSE,stringsAsFactors=FALSE)

table=cbind(data0[,1],data0[,2],paste(data0[,3],data0[,4],data0[,5],sep=":"))

temp0=paste(table[,1],table[,2],sep=":")

table=table[order(temp0),]

for(i in 2:length(list)){

data=read.table(list[i],header=FALSE,stringsAsFactors=FALSE)

temp=paste(data[,1],data[,2],sep=":")

temp0=paste(table[,1],table[,2],sep=":")

tempr=intersect(temp0,temp)

loc0=which(temp0%in%tempr)

loc=which(temp%in%tempr)

table=table[loc0,]

data=data[loc,]

temp0=paste(table[,1],table[,2],sep=":")

table=table[order(temp0),]

temp=paste(data[,1],data[,2],sep=":")

data=data[order(temp),]

table=cbind(table,paste(data[,3],data[,4],data[,5],sep=":"))

}

colnames(table)=c("chr","loc",gsub(".txt","",list))

## write.table(table,"expression_summary.txt",sep="\t",row.names=FALSE,col.names=TRUE,quote=FALSE)

## use above command to check the colum order as M F h1-m h1-f h2-m h2-f

## screen the SNPs with the effective counts and pure parent snp type

test1=apply(table,1,function(x){length(grep("N",x))==0})

table=table[which(test1),]

testofparent=function(x){

temp=x[3:8]

temp=unlist(strsplit(temp,":"))

loctemp=8*(0:5)+4

a=as.numeric(temp[loctemp+1])/as.numeric(temp[loctemp])

b=as.numeric(temp[loctemp+2])/as.numeric(temp[loctemp])

c=rbind(a,b)

result=min(apply(c,2,max))

if(temp[3]==temp[11]&temp[11]==temp[19]&temp[27]==temp[35]&temp[35]==temp[43]){}else{result=0}

result

}

parenttestresult=apply(table,1,testofparent)

table=table[which(parenttestresult>0.9),]

##write.table(table,"expression_summary_afterfilter.txt",sep="\t",row.names=FALSE,col.names=TRUE,quote=FALSE)

output=c("chr","loc","Mcns","Fcns","M1","M2","M3","F1","F2","F3","HM1","HM2","HM3","HF1","HF2","HF3\n")

cat(paste(output,collapse="\t"),file="final_expression.txt")

cat("##here're the errors in hybrid expression!\n",file="error_location")

for(i in 1:nrow(table)){

temp=unlist(strsplit(table[i,],":"))

r=temp[c(1,2,5,29)]

r=c(r,max(as.numeric(temp[7:8])),max(as.numeric(temp[15:16])),max(as.numeric(temp[23:24])),max(as.numeric(temp[31:32])),max(as.numeric(temp[39:40])),max(as.numeric(temp[47:48])))

temp1=unlist(strsplit(table[i,c(9,11,13)],":"))

exp1=temp1[c(5,6,13,14,21,22)]

names(exp1)=temp1[c(1,2,9,10,17,18)]

temp2=unlist(strsplit(table[i,c(10,12,14)],":"))

exp2=temp2[c(5,6,13,14,21,22)]

names(exp2)=temp2[c(1,2,9,10,17,18)]

if(length(c(exp1[which(names(exp1)==r[3])],exp1[which(names(exp1)==r[4])]))==6&&length(c(exp2[which(names(exp2)==r[3])],exp2[which(names(exp2)==r[4])]))==6&&r[3]!=r[4]){

tempr=rbind(as.numeric(c(exp1[which(names(exp1)==r[3])],exp1[which(names(exp1)==r[4])])),as.numeric(c(exp2[which(names(exp2)==r[3])],exp2[which(names(exp2)==r[4])])))

tempr2=apply(tempr,2,max)

r=c(r,tempr2)

cat(paste(r,collapse="\t"),file="final_expression.txt",sep="\n",append=TRUE)

}else{

cat(paste(table[i,],collapse="\t"),file="error_location",sep="\n",append=TRUE)

}

}

## two files were generated, "final_expression.txt" (read counts of snps in all samples) & "error_location"(some problems in hybrid expression).

library(readr)

exp0=as.data.frame(read_tsv("final_expression.txt"))

exp=exp0[,c(1,2)]

annot=read.table("Teatree.annot",sep=" ",stringsAsFactors=FALSE)

annotout=c()

for(i in 1:nrow(exp)){

sc=annot[which(annot[,1]==exp[i,1]),]

x=max(which(sc[,2]<exp[i,2]))

y=min(which(sc[,3]>exp[i,2]))

if(x==y){

annotout=c(annotout,sc[x,4])

}else{

annotout=c(annotout,"NA")

}

print(i)

}

write.table(cbind(exp0,annotout),"final_expression_annotation.txt",sep="\t",row.names=FALSE,col.names=TRUE,quote=FALSE)

length(table(annotout))-1

data=read.table("final_expression_annotation.txt",sep="\t",stringsAsFactors=FALSE,header=TRUE)

data=data[,c(-1,-2,-3,-4)]

data=data[which(data[,13]!="NA"),]

data=data[,c(13,1:12)]

colnames(data)[1]="Geneid"

data=data[order(data[,1]),]

genelist=names(table(data[,1]))

genexpr=c()

for(i in 1:length(genelist)){

temp=as.matrix(data[which(data[,1]==genelist[i]),-1])

temp2=round(apply(temp,2,mean),2)

genexpr=rbind(genexpr,c(genelist[i],temp2))

}

colnames(genexpr)=colnames(data)

write.table(genexpr,"gene_expression.txt",sep="\t",row.names=FALSE,col.names=TRUE,quote=FALSE)

## 5. Data normalization and DEGs detection, noticing the difference of repetition numbers (sample with poor correlation was removed) (R scripts)

library(edgeR)## edgeR package

x=read.table("gene_expression.txt",sep="\t",stringsAsFactors=FALSE,header=TRUE,row.names=1)

y=as.matrix(cbind(x[,2:6],(x[7:9]+x[10:12])))

librarysize=c()

Y=DGEList(count=y,lib.size=librarysize,group=c(1,1,2,2,2,3,3,3))

Y=calcNormFactors(Y)## TMM normalization

write.table(Y$sample,"norm_factors.txt",sep="\t",row.names=TRUE,col.names=TRUE,quote=FALSE)

nf=Y$sample[,2]*Y$sample[,3]

nf=c(nf,nf[6:8])

x=x[,-1]

for(i in 1:ncol(x)){

x[,i]=x[,i]*1000000/nf[i]

}

x=round(x,2)

write.table(x,"gene_expression_normalized.txt",sep="\t",row.names=TRUE,col.names=TRUE,quote=FALSE)

e=read.table("gene_expression_normalized.txt",header=TRUE,row.names=1)

e2=e[,1:8]

e2[,6:8]=e[,6:8]+e[,9:11]

boxplot(log2(e2))

d=as.dist(1-cor(e2))

plot(hclust(d,method="average"))

library(edgeR)

Y=DGEList(count=e2,group=c(1,1,2,2,2,3,3,3))#######

Y=estimateDisp(Y)

## Hybrid vs Female

et=exactTest(Y,pair=c(2,3))

t=et$table

t=cbind(t,p.adjust(t[,3],method="fdr"))## False discovery rate adjusting

DE=rep("NS",nrow(t))

for(i in 1:nrow(t)){

if(t[i,1]>=log2(1.25)&&t[i,4]<0.05){DE[i]="Up"}

if(t[i,1]<=-log2(1.25)&&t[i,4]<0.05){DE[i]="Down"}

}## fold-change > 1.25 and p-value < 0.05

t=cbind(t,DE)

write.table(t,"DEGslistH_F.txt",sep="\t",row.names=TRUE,col.names=TRUE,quote=FALSE)

## Female vs Male

et=exactTest(Y,pair=c(1,2))

t=et$table

t=cbind(t,p.adjust(t[,3],method="fdr"))## False discovery rate adjusting

DE=rep("NS",nrow(t))

for(i in 1:nrow(t)){

if(t[i,1]>=log2(1.25)&&t[i,4]<0.05){DE[i]="Up"}

if(t[i,1]<=-log2(1.25)&&t[i,4]<0.05){DE[i]="Down"}

}## fold-change > 1.25 and p-value < 0.05

t=cbind(t,DE)

write.table(t,"DEGslistF_M.txt",sep="\t",row.names=TRUE,col.names=TRUE,quote=FALSE)

## Hybrid vs Male

et=exactTest(Y,pair=c(1,3))

t=et$table

t=cbind(t,p.adjust(t[,3],method="fdr"))## False discovery rate adjusting

DE=rep("NS",nrow(t))

for(i in 1:nrow(t)){

if(t[i,1]>=log2(1.25)&&t[i,4]<0.05){DE[i]="Up"}

if(t[i,1]<=-log2(1.25)&&t[i,4]<0.05){DE[i]="Down"}

}## fold-change > 1.25 and p-value < 0.05

t=cbind(t,DE)

write.table(t,"DEGslistH_M.txt",sep="\t",row.names=TRUE,col.names=TRUE,quote=FALSE)

## 6. Cis- and trans- detection (R scripts)

library(car)

e=read.table("gene_expression_normalized.txt",header=TRUE,row.names=1)

result2=c()

for(i in 1:nrow(e)){

temp=as.numeric(e[i,])

temp2=round(c(mean(temp[1:2]),mean(temp[3:5]),mean(temp[6:8]),mean(temp[9:11])),0)

B=binom.test(temp2[3],temp2[3]+temp2[4],0.5)$"p.value"

A=fisher.test(matrix(temp2,nrow=2))$"p.value"

C=binom.test(temp2[1],temp2[2]+temp2[1],0.5)$"p.value"

result2=rbind(result2,c(rownames(e)[i],A,B,C))

}

## False discovery rate adjusting

fdra=p.adjust(result2[,2],method="fdr")

fdrb=p.adjust(result2[,3],method="fdr")

fdrc=p.adjust(result2[,4],method="fdr")

result2=cbind(result2,fdra,fdrb,fdrc)

colnames(result2)=c("geneid","p(A=B)","p(B=1)","p(A=1)","fdr(A=B)","fdr(B=1)","fdr(A=1)")

write.table(result2,"AB_result.txt",sep="\t",row.names=FALSE,col.names=TRUE,quote=FALSE)

|  | Sample | Total Clean Reads (Mb) | Total Clean Bases (Gb) | Clean Reads Q20 (%) | Clean Reads Q30 (%) |
| --- | --- | --- | --- | --- | --- |
| *Camellia chekiangoleosa* | *che*2_2 | 44.37 | 6.66 | 97.24 | 92.47 |
|  | *che*3_2 | 44.81 | 6.72 | 97.35 | 92.72 |
|  | *che*4_2 | 44.98 | 6.75 | 97.38 | 92.80 |
| *C. azalea* | *aze*4_1 | 44.85 | 6.73 | 97.33 | 92.70 |
|  | *aze*5_2 | 44.54 | 6.68 | 97.16 | 92.31 |
|  | *aze*6_2 | 42.93 | 6.44 | 97.36 | 92.75 |
| *C. amplexicaulis* | *amp*1_1 | 42.61 | 6.39 | 97.34 | 92.66 |
|  | *amp*2_2 | 44.41 | 6.66 | 97.35 | 92.74 |
|  | *amp*3_2 | 43.03 | 6.45 | 97.47 | 93.03 |
| *aza* × *che* | *aze*_*che* 1A | 44.13 | 6.62 | 97.66 | 93.46 |
|  | *aze*_*che* 3A | 44.87 | 6.73 | 97.65 | 93.47 |
|  | *aze*_*che* 7A | 44.54 | 6.68 | 97.62 | 93.39 |
| *aza* × *amp* | *aze*_*amp* 13A | 44.33 | 6.65 | 97.55 | 93.22 |
|  | *aze*_*amp* 47A | 45.04 | 6.76 | 97.52 | 93.12 |
|  | *aze*_*amp* 53A | 45.16 | 6.77 | 97.56 | 93.23 |

**Additional file 1: Table S1.** The sequencing results of different accessions in this study

Note: *aza* × *che*, F_1_ hybrid of *Camellia azalea* × *C. chekiangoleosa*; *aza* × *amp*, F_1_ hybrid of *C. azalea* × *C. amplexicaulis*.

| Classification | Binomial test | Binomial test | Fisher’s test | Additional criteria |
| --- | --- | --- | --- | --- |
| Conserved | P1=P2 | A1=A2 | P1/P2=A1/A2 | NA |
| *cis* only | P1≠P2 | A1≠A2 | P1/P2=A1/A2 | NA |
| *trans* only | P1≠P2 | A1=A2 | P1/P2≠A1/A2 | NA |
| *cis+trans* | P1≠P2 | A1≠A2 | P1/P2≠A1/A2 | Log_2_(P1/P2)/log_2_(A1/A2) > 1 |
| *cis×trans* | P1≠P2 | A1≠A2 | P1/P2≠A1/A2 | Log_2_(P1/P2)/log_2_(A1/A2) < 1 |
| Compensatory | P1=P2 | A1≠A2 | P1/P2≠A1/A2 | NA |
| Ambiguous | P1≠P2 | A1=A2 | P1/P2=A1/A2 | NA |
| Ambiguous | P1=P2 | A1≠A2 | P1/P2=A1/A2 | NA |
| Ambiguous | P1=P2 | A1=A2 | P1/P2≠A1/A2 | NA |

**Additional file 1: Table S2.** Classification of different regulatory types.

Note: P1/2, Parent 1/2; A1, allele form P1in hybrid; A2, allele from P2 in hybrid.

**Additional file 1: Table S3.** Sequences used for genetic distance analysis in this study.

| No. | Gene | Taxon | Accession Number |
| --- | --- | --- | --- |
| 1 | ITS | *Camellia amplexicaulis* | EU579676 |
| 2 | ITS | *Camellia azalea* | EU579681 |
| 3 | ITS | *Camellia chekiangoleosa* | EU579686 |
| 4 | ITS | *Camellia sinensis* | FJ432121 |
| 5 | ITS | *Coffea canephora* | DQ153593 |
| 6 | ITS | *Coffea eugenioides* | DQ153588 |
| 7 | ITS | *Arabidopsis thaliana* | AJ232900 |
| 8 | ITS | *Arabidopsis arenosa* | U43232 |
| 9 | *AMYREL* | *Drosophila melanogaster* | AF022713 |
| 10 | *AMYREL* | *Drosophila sechellia* | AF039558 |

Note: Genetic distance were evaluated using the Mega 6.0 software with the Kimura 2-parameter model.

**
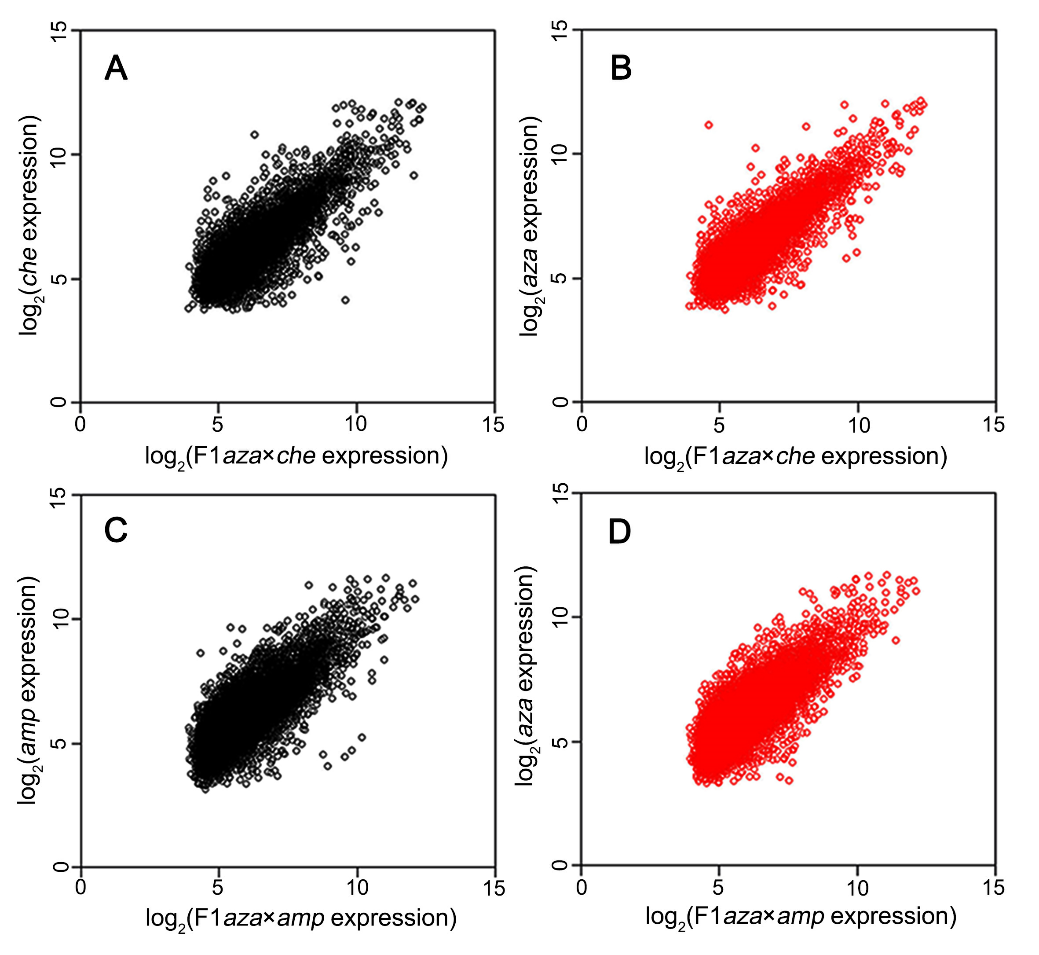
**

**Additional file 1: Figure S1.** Plots compare total expression levels of F_1_ hybrids to total expression levels of parental species. *aza*, *Camellia azalea*; *che*, *C. chekiangoleosa*; *amp*, *C. amplexicaulis*; F1*aza×che*, F_1_ hybrid of *C. azalea* × *C. chekiangoleosa*; F1*aza×amp*, F_1_ hybrid of *C. azalea* × *C. amplexicaulis*.

**
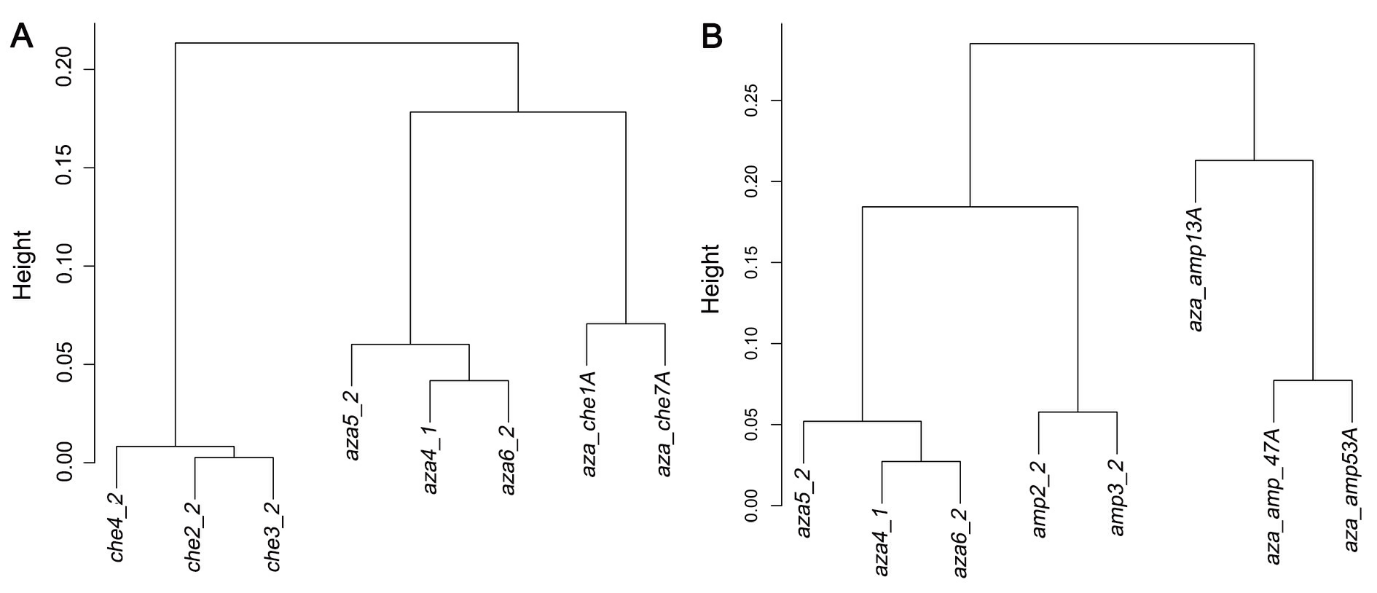
**

**Additional file 1: Figure S2.** Clustering analysis showing the repeatability of biological replicates for each species and the hybrid. **(A)** The cross of *Camellia azalea* × *C. chekiangoleosa*. **(B)** The cross of *Camellia azalea* × *C. amplexicaulis*. Samples started with *aza*, *C. azalea*; *che*, *C. chekiangoleosa*; *amp*, *C. amplexicaulis*. *aza_che*, F_1_ hybrid of *Camellia azalea* × *C. chekiangoleosa*; *aza*_*amp*, F_1_ hybrid of *Camellia azalea* × *C. amplexicaulis*.
